# Supplementary material for: Fast Recombination of Charge-Transfer State in Organic Photovoltaic Composite of P3HT and Semiconducting Carbon Nanotubes Is the Reason for Its Poor Photovoltaic Performance
Source: Int J Mol Sci. 2023 Feb 17;24(4):4098. doi: 10.3390/ijms24044098 (PMC9961616; doi:10.3390/ijms24044098)
Supplement: Supplementary file 1 [file ijms-24-04098-s001.zip › ijms-2157265-supplementary.pdf]

Supporting Information

Fast recombination of charge-transfer state in organic photovoltaic composite of P3HT and semiconducting carbon nanotubes is the reason of its poor photovoltaic performance

Mikhail N. Uvarov[1], Elena S. Kobeleva[1], Konstantin M. Degtyarenko[2],

Vladimir A. Zinoviev[3], Alexander A. Popov[1,4], Evgeny A. Mostovich[4], Leonid V. Kulik[1]

[1] Voevodsky Institute of Chemical Kinetics and Combustion of the Siberian Branch of the Russian Academy of Sciences, Novosibirsk, Russia

[2] Siberian Physico-Technical Institute, Tomsk State University, Russia

[3] Rzhanov Institute of Semiconductor Physics of the Siberian Branch of the Russian Academy of Sciences, Novosibirsk, Russia

[4]Novosibirsk State University, Novosibirsk, Russia

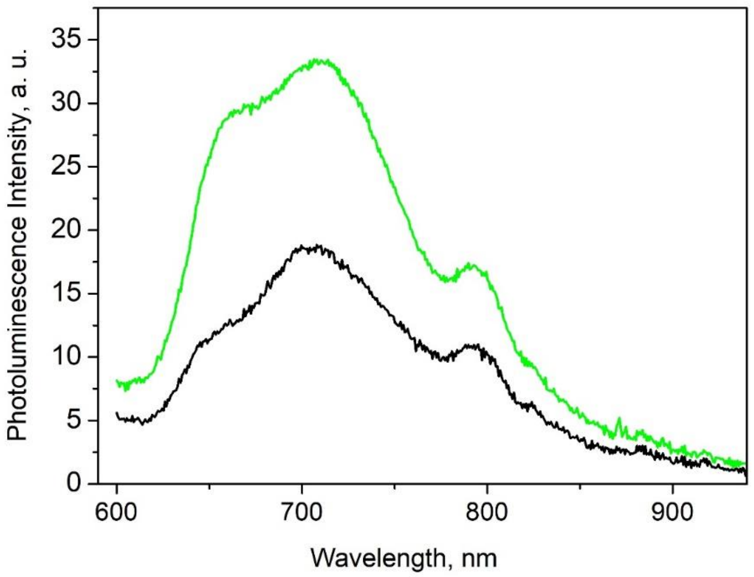

Figure S1. Photoluminescence spectra of pristine P3HT (green line) and P3HT/s-SWCNT composite with 2 % weight fraction of s-SWCNT (black line). The excitation at 525 nm was performed by tunable Xenon light source TLS300XU (Newport). The films were spin-coated on quartz substrate from o-dichlorobenzene solution at 2000 rpm. The shoulder at 790 nm is an experimental artefact.

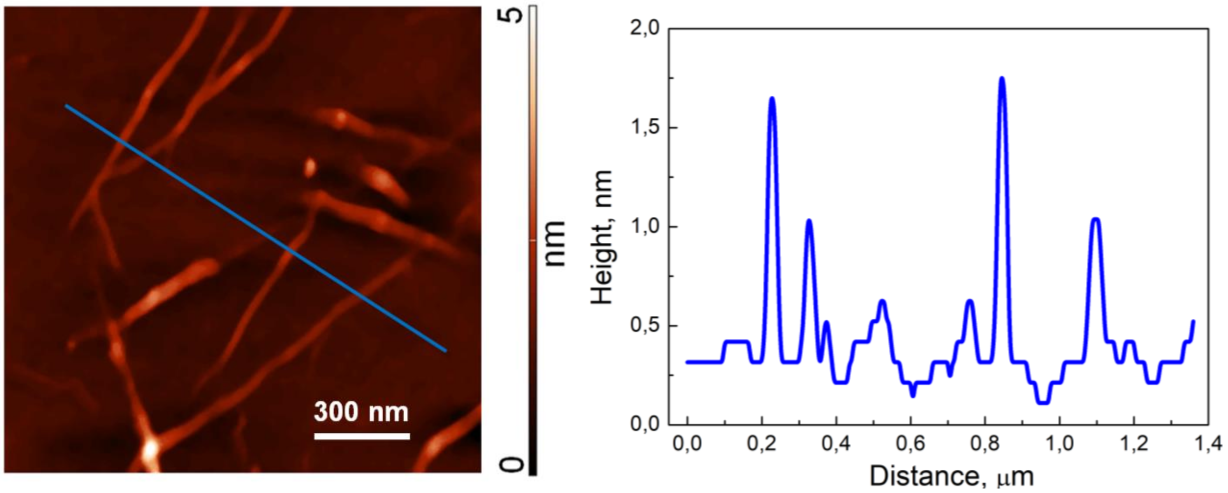

Figure S2. Left panel: AFM image of IsoNanotube-S s-SWCNTs spin-coated from o-dichlorobenzene dispersion (prepared by sonication) onto silicon plate at 8000 rpm. Right panel: height profile of this image along the blue line at the left panel.
